# Unraveling the Interconnections Between Statistical Learning and Dyslexia: A Review of Recent Empirical Studies

**DOI:** 10.3389/fnhum.2021.734179

**Published:** 2021-10-22

**Authors:** Sonia Singh, Christopher M. Conway

**Affiliations:** ^1^Callier Center for Communication Disorders, School of Behavioral and Brain Sciences, The University of Texas at Dallas, Dallas, TX, United States; ^2^Brain, Learning, and Language Lab, Center for Childhood Deafness, Language, and Learning, Boys Town National Research Hospital, Omaha, NE, United States

**Keywords:** reading, developmental dyslexia (DD), statistical learning, implicit learning, developmental language disorder (DLD)

## Abstract

One important aspect of human cognition involves the learning of structured information encountered in our environment, a phenomenon known as statistical learning. A growing body of research suggests that learning to read print is partially guided by learning the statistical contingencies existing between the letters within a word, and also between the letters and sounds to which the letters refer. Research also suggests that impairments to statistical learning ability may at least partially explain the difficulties experienced by individuals diagnosed with dyslexia. However, the findings regarding impaired learning are not consistent, perhaps partly due to the varied use of methodologies across studies – such as differences in the learning paradigms, stimuli used, and the way that learning is assessed – as well as differences in participant samples such as age and extent of the learning disorder. In this review, we attempt to examine the purported link between statistical learning and dyslexia by assessing a set of the most recent and relevant studies in both adults and children. Based on this review, we conclude that although there is some evidence for a statistical learning impairment in adults with dyslexia, the evidence for an impairment in children is much weaker. We discuss several suggestive trends that emerge from our examination of the research, such as issues related to task heterogeneity, possible age effects, the role of publication bias, and other suggestions for future research such as the use of neural measures and a need to better understand how statistical learning changes across typical development. We conclude that no current theoretical framework of dyslexia fully captures the extant research findings on statistical learning.

## Introduction

An important question is whether dyslexia is characterized by deficits specific only to reading and language or if the learning difficulties are more global in nature. One area of research that can help answer this question is focused on the phenomenon of statistical learning, which is a neurocognitive process that involves the extraction of statistically based patterns from the environment (Saffran et al., 1996; Saffran, 2003; Turk-Browne, 2012; Emberson and Rubinstein, 2016; Armstrong et al., 2017; Batterink and Paller, 2017; Christiansen, 2019; Siegelman et al., 2019; Conway, 2020). Because language (both spoken and written) is a heavily patterned input domain, statistical learning is believed to be important, and perhaps even necessary, for successful language learning (Conway and Pisoni, 2008; Conway et al., 2010; Romberg and Saffran, 2010; Arciuli and von Koss Torkildsen, 2012; Christiansen et al., 2012; Lammertink et al., 2017; Seidenberg and MacDonald, 2018). For instance, statistical learning has been tied to spelling ability (Ise et al., 2012; Chetail, 2017; Treiman, 2018; Tong et al., 2019), knowledge of syntax (Arciuli and Simpson, 2012; Kidd, 2012; Petersson et al., 2012), spoken sentence processing (Conway et al., 2010), and reading (Sperling et al., 2004; Arciuli and Simpson, 2012; Treiman et al., 2014; Staels and van den Broeck, 2017; Arciuli, 2018; Banai and Ahissar, 2018; Schmalz et al., 2018; Sawi and Rueckl, 2019). Furthermore, atypical statistical learning has also been implicated in both spoken and written language difficulties (Lum et al., 2013; Krishnan et al., 2016; van Witteloostuijn et al., 2017; Arciuli and Conway, 2018; Bogaerts et al., 2020). Therefore, because learning spoken and written language appears to be supported by statistical learning processes in typical development, it is a distinct possibility that some language disorders – including dyslexia – might arise from a deficit in or irregularities with statistical learning.

In this review, we examine the evidence exploring a possible link between atypical statistical learning and dyslexia. Some studies have demonstrated intact learning in individuals with dyslexia, whereas a number of others have identified impairment. It is therefore not entirely clear to what extent statistical learning impairments are associated with dyslexia. In fact, at least one recent review concluded that there was insufficient evidence regarding the statistical learning – dyslexia link (Schmalz et al., 2017). One reason for the discrepant findings is that they may be due to the multi-component nature of statistical learning (e.g., Daltrozzo and Conway, 2014; Arciuli, 2017; Arciuli and Conway, 2018; Bogaerts et al., 2020; Conway, 2020). That is, different tasks or paradigms may be tapping into different components or sub-processes that underlie this learning ability, with dyslexia perhaps being associated with difficulties for only a subset of these tasks. In addition, a statistical learning deficit may not be consistent for all individuals with dyslexia; factors such as age, gender, extent of learning deficit, presence or absence of other comorbidities, and native language may all affect the statistical learning-dyslexia link.

Thus, in this review, we pay particularly close attention to the differences across paradigms and methodologies used in order to better understand the nature of a statistical learning deficit in dyslexia, if it exists. Because there have been recent meta-reviews and literature reviews on this growing area of research (Lum et al., 2013; Schmalz et al., 2017; van Witteloostuijn et al., 2017; Sawi and Rueckl, 2019; Bogaerts et al., 2020), our review is focused almost exclusively on the most recent studies that were not included or discussed in these prior reviews. In the remainder of this paper, first, we review the most common theories that have been leveraged to explain the core deficits of dyslexia. Next, we provide an overview of the phenomenon of statistical learning and review three recent meta-analyses/reviews that have examined statistical learning in dyslexia. Then, we review the most recent research examining statistical learning in adults and children with dyslexia and offer suggestive trends that emerge from this review. Finally, we return to the theoretical frameworks and assess whether or not they are adequate for capturing the existing research findings related to statistical learning and dyslexia.

## Dyslexia: Theoretical Frameworks

### Dyslexia: A Brief Overview

Prior to the reorganization of diagnostic criteria in the DSM-5 (American Psychiatric Association, 2013), ‘dyslexia’ meant an array of impediments characterized by poor word recognition, decoding, and spelling abilities (i.e., difficulty in ‘learning to read’). Additionally, specific subtypes of dyslexia were also thought to exist, depending on severity of the disability. With little consensus over these complex subtypes, the disorder eventually became housed under a broader classification that focused more on both the heterogeneity within dyslexia as well as its co-occurrence with other disorders (Snowling et al., 2020). Currently, developmental dyslexia is classified under ‘developmental disorders’ (Ullman et al., 2020); and to be included within this category an individual must experience difficulties in various aspects of language learning (Bishop, 2017) such as learning to read and spell. Snowling et al. (2020) argue that dyslexia should be considered as less of a purely reading disorder and more of a persistent difficulty with decoding and spelling fluency, markedly affecting academic performance. Thus, rather than referring to a specific reading disorder, the classification of ‘dyslexia’ henceforth reflects these updates. Dyslexia is often comorbid with other cognitive disabilities such as arithmetic learning disability (von Aster and Shalev, 2007; Dirks et al., 2008; Landerl and Moll, 2010), attention deficit hyperactivity disorder (Gilger et al., 1992; Pennington, 2005), specific speech disorder (Pennington and Bishop, 2009), developmental language disorders (Catts et al., 2005; Snowling et al., 2020) and even developmental coordination disorder (Kaplan et al., 1998). This suggests that reading ability is heavily intertwined with other aspects of cognition and academic skills required for typical development as has been recently outlined by Moll et al. (2020). These patterns of comorbidity raise the possibility that at least for some individuals, the problems are due to more general issues with attention, learning, or other cognitive processes that span across these different domains. Different patterns of comorbidities may complicate the interpretation of different studies that assess statistical learning in individuals with dyslexia.

**FIGURE 1:**
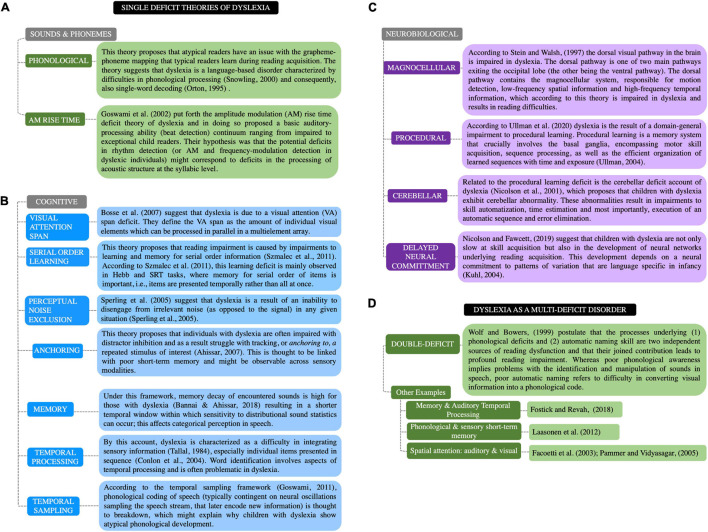
Theoretical framework for dyslexia. **(A–C)** Single deficit theories of dyslexia. **(D)** Multiple deficit theories of dyslexia. The single deficit theories are clustered by category [**(A)** sounds and phonemes; **(B)** cognitive; **(C)** neurobiological]. Each theory is appropriately labeled (left); each is accompanied by a brief description (right).

To better understand how and why statistical learning deficits might underlie developmental dyslexia next, we review prominent deficit-centered theories of dyslexia and their relevance to statistical learning. By reading through the framework below it is apparent that some of these theories are consistent with the notion of more general and pervasive cognitive deficits. Figure 1 summarizes these theories and is organized into four categories based on the postulated type of deficit: sounds and phonemes, cognitive, neurobiological, and multiple deficit theories.

### Single Deficit Theories Related to Sounds and Phonemes

First, considering theories related to sounds and phonemes (Figure 1A), the phonological deficit theory is perhaps the most prominent perspective (Vellutino et al., 2004). Here decoding involves analyzing printed symbol forms (e.g., letters) and mapping them to the sounds to which they refer (e.g., that the word ‘pot’ begins with a /p/ sound; Castles et al., 2018). Under this view, reading impairment results from a disruption in the grapheme-phoneme mapping learned during typical reading acquisition. However, in recent years, the existence of a purely phonological deficit has been challenged (Banai and Ahissar, 2018; Cabbage et al., 2018; Lachmann, 2018; Giofrè et al., 2019). Instead, other sound-related theories such as the amplitude modulation rise-time deficit theory (Goswami et al., 2002) have been proposed.

If dyslexia is at its core a problem with processing sounds and phonemes, as these two theories suggest, then we might expect a bigger impairment in dyslexia for statistical learning tasks using auditory and phonological-based stimuli compared to non-auditory or non-phonological stimuli. Accordingly, statistical learning tasks involving visual or visual-motor patterns would be expected to be less affected. If such a result was found, then it would be difficult to conclude that dyslexia is associated with domain-general statistical learning impairments but rather is a sound or phoneme-based disorder as this group of theories imply.

### Single Deficit: Cognitive Theories

Some other theories focus on more general cognitive processes (Figure 1B). These theories propose impairments in visual attention (Bosse et al., 2007), serial order learning (Szmalec et al., 2011), temporal processing and sampling (Tallal, 1984; Goswami, 2011), memory, and the ability to track and/or disengage from environmental stimuli (Sperling et al., 2005; Ahissar, 2007). These theories therefore suggest that difficulties with such cognitive processes have downstream effects on the ability to learn to read.

Interestingly, the deficits described in this section have been found across sensory modalities. For instance, the attention span deficit is not limited to the visual domain as an auditory attention deficit has also been observed in adults with dyslexia (see Hari and Renvall, 2001). Additionally, although Sperling et al. (2005) observed reduced perceptual discrimination in the visual domain, similar degraded sensitivity has also been found while tracking sound statistics (Banai and Ahissar, 2018). Most of these theories also seem to relate relatively straightforwardly to statistical learning, with any of these proposed cognitive deficits likely leading to impaired performance on statistical learning tasks. For instance, a deficit of attention, serial order learning, or temporal sampling all could affect statistical learning (at least for those tasks involving serial order or temporal input). Therefore, these theoretical positions provide a rationale not only for poor reading performance but difficulties with statistical learning, explaining any association that might exist between the two.

### Single Deficit: Neurobiological Theories

Another set of theories emphasize limitations in neural function or disturbances to specific neural circuits (Figure 1C), such as the magnocellular system (Stein and Walsh, 1997), the basal ganglia and related circuitry underlying procedural memory (Ullman, 2004; Ullman et al., 2020), and/or the cerebellum (Nicolson et al., 2001). More recently, Nicolson and Fawcett (2019) have suggested that a delay in the development of neural networks associated with communication (e.g., hearing, speech) could eventually cascade into several issues that characterize dyslexia (e.g., phonological circuits that are less organized, a difficulty in automaticity of skills, etc.).

Like the cognitive theories, these neural theories appear to be closely related to statistical learning. Deficits to the magnocellular system and cerebellar and procedural memory systems could all negatively affect statistical learning abilities. On the other hand, each of these theories would provide slightly different predictions for how a statistical learning deficit might play out. Although procedural memory is sometimes thought to be synonymous with statistical learning, the former has a more specific definition focused on the basal ganglia circuitry (Ullman et al., 2020) whereas the latter appears to involve multiple brain circuits such as posterior perceptual networks, anterior brain areas including the prefrontal cortex, in addition to subcortical structures (Frost et al., 2015; Sawi and Rueckl, 2019; Conway, 2020). So, too many tasks considered to measure statistical learning do not necessarily tap into procedural memory; one task that has been implicated as a procedural memory task based on neuroimaging data is the serial-reaction time task (Ullman et al., 2020). Therefore, if deficits on this specific task are found in individuals with dyslexia, then this would be consistent with the procedural memory deficit hypothesis. On the other hand, if deficits are found on other statistical learning tasks that are not thought to tap into procedural memory specifically, such as the embedded patterns task, then this would be indicative of a more general statistical learning deficit. These various tasks will be described in more detail in Section “Statistical Learning.”

### Multiple Deficit Theories

All of the previous theories focused mainly on a particular single deficit to account for reading impairment. However, as dyslexia is considered a complex disorder with a multifactorial etiology, it is possible that multi-deficit models will provide improved explanatory power (Elliott and Grigorenko, 2014). Within the multiple deficit model framework, symptoms of complex developmental disorders are thought to be a result of an incremental and interconnected set of dysfunctional processes (Banaschewski and Rohde, 2008; Moll et al., 2020). The multiple deficit theories tend to integrate pre-existing single deficit theories; some examples of this are listed in Figure 1D.

In sum, one common thread among both single and multiple deficit theories is that several of them are focused on difficulties with learning or processing items in a temporal sequence, regardless of whether the input consists of language material (e.g., temporal processing deficit, SOLID, anchoring deficit, procedural learning). Thus, based on a sampling of these deficit theories, it would not be surprising to find that dyslexia is associated with a generalized statistical learning deficit, though as discussed earlier, certain theoretical frameworks provide nuanced views of how such a learning deficit would be manifested. Next, we outline the construct of statistical learning and some common ways that it is measured in the lab.

## Statistical Learning

Statistical learning can be defined as the (incidental) learning of structured input patterns encountered in the environment (Conway, 2020). Such input includes the regularities found in both spoken and written language. Historically, there are several research areas that arguably all relate to the construct of statistical learning. For instance, before the term ‘statistical learning’ became widely used, there was a long history of research focused on ‘implicit learning’ (Reber, 1967; see Box 1), generally defined as incidental learning that occurs without conscious awareness (Cleeremans et al., 1998). Similarly, ‘sequence learning’ has been a term used to describe the learning of input sequences specifically – as opposed to learning patterns in a spatial array – such as a repeating fixed sequence (e.g., Nissen and Bullemer, 1987). The stance taken here is that statistical learning is an umbrella term used to describe the learning of any type of pattern, whether it be a sequence or a spatial array, across any perceptual or motor modality, and one that can occur implicitly or explicitly, depending on the task, learning context, or individual (Conway, 2020).

**Box 1. Multiple Systems for Statistical Learning.**

Perruchet and Pacton (2006) proposed that ‘statistical learning’ and ‘implicit learning’ might be conceptualized as two approaches underlying one phenomenon, a type of domain-general incidental learning. However, despite this potential overlap, it is likely that there is not a single form of learning but rather multiple learning mechanisms that might each be associated with unique effects as elicited by particular task paradigms. For instance, Dehaene et al. (2015) outlined evidence to suggest that the learning of sequence patterns involves a number of distinct processes for acquiring different types of knowledge: transition and timing, chunking, ordinal information, algebraic patterns, and nested structures. Dehaene et al. (2015) furthermore state that several of these learning processes may be recruited during any single paradigm or learning episode, with the type of learning matching the task requirements. According to another theory, statistical learning is based on two primary neurocognitive mechanisms functioning in tandem: 1) a bottom-up, perceptual-based “suite” of learning mechanisms that are automatic and operate independently of attention and 2) a top-down process that is attention-dependent and can modulate learning by focusing attention on certain stimuli or aspects of the input (Conway, 2020).

The difficulty with such a broad umbrella construct is that it can be measured in many different ways. This leads to potential problems, because although two different tasks might purportedly both measure statistical learning, each one may emphasize a slightly different type or aspect of learning, which is not always openly acknowledged or even fully understood. This in turn can lead to discrepancies across studies: two different studies assessing statistical learning in a developmental disorder such as dyslexia might find inconsistent results (that is, one study might show intact learning in dyslexia whereas the other shows impaired learning) but this in turn might be due to the use of different tasks (an issue pointed out by Arciuli and Conway, 2018 and Bogaerts et al., 2020).

**FIGURE 2:**
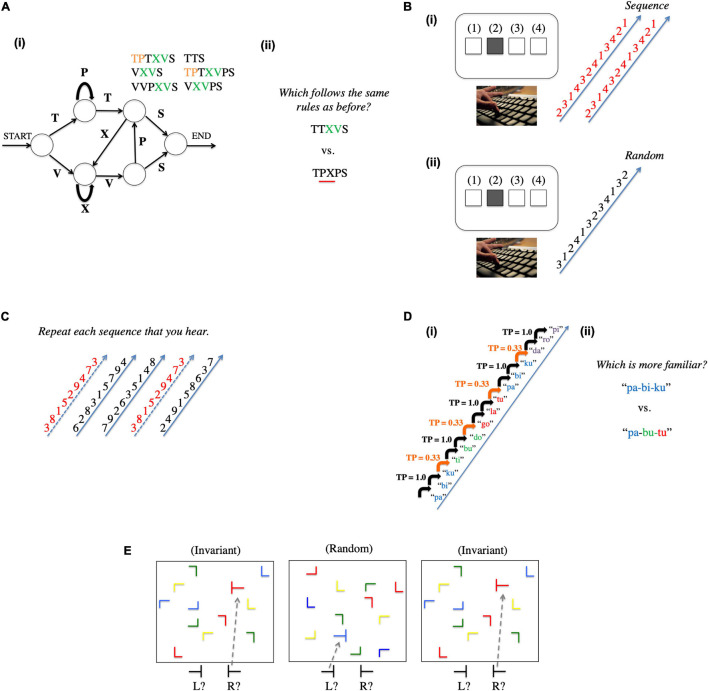
Figure showing frequently used statistical learning tasks. **(A)** Artificial grammar learning task (Reber, 1967). (i) In this paradigm, participants are exposed to stimulus sequences generated from an artificial grammar that dictates the order that particular stimuli can occur in the sequence. (ii) Following exposure, participants make grammaticality judgments on novel sequences that either are produced by the same grammar or contain violations of the grammatical rules. **(B)** Serial reaction time task (Nissen and Bullemer, 1987). (i) In this paradigm, participants view stimuli occurring in one of four locations; on each trial, they are instructed to press a button corresponding to that particular location as quickly as possible. The stimuli appear in a sequence that repeats itself. (ii) Learning is assessed by comparing reaction times to the repeating sequence compared to stimuli that appear in random order. **(C)** Hebb repetition task (Hebb, 1961). Participants are presented with a sequence of stimuli and must repeat back the sequence in the correct order. On some trials, the sequence is random (solid arrows) and on other trials the sequence is a repetition (dashed arrows) from a previous sequence. Learning is assessed by comparing immediate serial recall for the repeating sequence compared to random sequences. **(D)** Embedded pattern (originally called *word segmentation* or *triplet*) task (Saffran et al., 1996). (i) In the typical version of this paradigm, participants are provided with passive exposure to stimuli that conform to 3-syllable “words”; (ii) Following familiarization, participants are given a two-alternative forced-choice test, judging which of two 3-syllable sequences are “familiar.” Sequences can only be distinguished based on differences in transitional probabilities among the syllables (i.e., whether they conform to the “words” presented in the familiarization stream). **(E)** Contextual cueing task (Chun and Jiang, 1998). On each trial, participants view a scene with different colored visual stimuli arranged on the screen and must press one of two buttons as quickly as possible to indicate whether a target stimulus (in this case a “T” on its side) is pointed to the left or right. On some trials, the background stimuli occur in the same invariant arrangement, and on other trials, the background stimuli are presented in random arrangements. Learning is assessed by quicker responses to targets in the invariant scenes compared to random scenes (adapted from Arciuli and Conway, 2018).

Despite these issues, statistical learning tasks generally have a common component: they involve presentation of patterned input under (usually) incidental learning conditions, resulting in learning that may or may not be accompanied by conscious awareness. Here, we describe five commonly used tasks to measure statistical learning: the artificial grammar learning task (Reber, 1967), serial reaction time task (Nissen and Bullemer, 1987), Hebb repetition learning paradigm (Hebb, 1961), the embedded pattern task (Saffran et al., 1996), and the contextual cueing task (Chun and Jiang, 1998). These tasks are depicted in Figure 2 (adapted from Arciuli and Conway, 2018) and described below.

First, Reber (1967) conducted foundational studies using an artificial grammar learning (AGL) paradigm to investigate whether people could learn the patterns presented within letter strings simply by exposure to those stimuli. In this task there is a familiarization phase involving exposure to patterns generated from an artificial grammar. A test phase then follows in which participants are prompted to classify novel input patterns as grammatical or not. In such an unsupervised learning environment it was found that learners could deduce pattern information from previously encountered stimuli to make grammaticality judgments regarding novel stimuli, despite a general inability to verbally articulate what they had learned. Artificial grammar learning became an established tool used to probe implicit learning abilities (Reber, 1967; Dienes et al., 1991). Artificial grammar learning performance is sometimes measured in terms of two types of knowledge, namely, ‘grammaticality’ and ‘chunk strength’ (Knowlton and Squire, 1996). Grammaticality is the extent to which the training stimuli comply with the rules of the artificial grammar employed, such as letter positional information. Chunk strength refers to whether a test item is composed of previously encountered letter fragments in the training phase, generally corresponding to item similarity (see also, Cleeremans et al., 1998). Although these two types of information overlap, stimuli can be designed to separate out the influence of both on test performance (Knowlton and Squire, 1996).

A second paradigm called the serial reaction time (SRT) task has also frequently been used to study implicit pattern learning (Nissen and Bullemer, 1987; Robertson, 2007). In this task participants are presented with a repeating fixed sequence and must track the occurrence of each stimulus with a specific response choice (via button press). Participants remain uninformed about the nature of the repeating sequence. Participant response times (RTs) decrease with exposure to the repeating sequence relative to random sequences, indicating sequence-specific learning. The SRT task is known to reflect procedural memory specifically (i.e., learning and memory rooted in basal ganglia circuits; Ullman et al., 2020).

A third paradigm relies upon what is referred to as the Hebb repetition effect (Hebb, 1961; Szmalec et al., 2011; Henderson and Warmington, 2017). A Hebb learning task involves presentation of an ordered sequence repeatedly (typically nonsense syllables), similar to a language learner encountering new words over multiple exposures. Note that this is not dissimilar to the SRT task described above. However, rather than pressing a button after each individual item in the sequence, the participant’s task is to repeat each sequence (typically verbally) after it is presented. Over time, recall for the repeated Hebb sequence improves relative to filler (non-repeated) sequences, indicating learning. Awareness is not necessary for learning in this task and is therefore considered implicit (McKelvie, 1987) at least in part. Performance on Hebb repetition learning has been strongly linked to novel word learning. This is because word learning is thought to be incumbent on the same mechanism responsible for ordering sequences of phonological information, or short-term serial order memory (Szmalec et al., 2009). Additionally, learned Hebb sequences are thought to enter long-term memory as novel lexical forms. This is evident because syllable sequences acquired in this task are known to compete with existing word-forms in the lexicon (Szmalec et al., 2009).

Another important paradigm was later introduced by Saffran et al. (1996), referred to here as the embedded pattern task, whose landmark study began the field of statistical learning in earnest (for a recent review, see Saffran and Kirkham, 2018). They demonstrated that 8-month-old infants were capable of using sequential statistics to extract information regarding word boundaries in an artificial language composed of nonsense words presented as continuous speech (e.g., ‘bidakupadotigolabubidaku’). The idea is that once infants learn the transitional probabilities embedded in the speech stream, they can infer where the word boundaries are (example, ‘pretty#baby’). Furthermore, they found that infants could discriminate between word (e.g., ‘pretty’) and part-word (e.g., ‘ty#ba’) stimuli with longer listening times for part-words. This statistical learning phenomenon has been demonstrated not only with speech-like input, but also for non-linguistic sound sequences (Saffran et al., 1999) and visual scenes (Fiser and Aslin, 2001, 2002).

Finally, apart from the ‘sequence-oriented’ tasks described above, ‘non-sequential’ tasks (involving learning statistical regularities that are not sequentially presented) have also been used. In the contextual cueing task, for instance, participants are instructed to detect a target amidst a complex visual display. On some trials the background context features offer a reliable cue to the target location. Over time, participants are able detect the target faster on these consistent context trials compared to trials without the contextual cues (Chun and Jiang, 1998).

These five tasks make up the vast majority of studies that form the focus of this review. To serve as a foundation of our empirical review, we turn to the recent metanalytical studies and reviews that have examined whether dyslexia is associated with impairment to statistical learning.

## Statistical Learning in Dyslexia: Recent Meta-Analyses and Systematic Reviews

Several recent metanalyses and literature reviews have focused on statistical learning in those with dyslexia (Lum et al., 2013; Schmalz et al., 2017; van Witteloostuijn et al., 2017; see also Sawi and Rueckl, 2019; Bogaerts et al., 2020) thus providing a systematic evaluation of learning by task paradigm. For this reason, we first briefly review these relevant comprehensive works: one focusing on the SRT task (Lum et al., 2013) another on the artificial grammar learning task (van Witteloostuijn et al., 2017), and yet another that included both the SRT and artificial grammar learning tasks (Schmalz et al., 2017).

Lum et al. (2013) provided a formal meta-analysis of 14 studies that examined SRT learning in individuals with and without dyslexia. Their results showed evidence of a learning impairment in those with dyslexia compared to controls, with an effect size of 0.449. Interestingly, Lum et al. (2013) also found that effect sizes were smaller for older participants who were assessed on SRT tasks that incorporated either second-order conditional statistics or greater numbers of exposures to the stimuli. Lum et al. (2013) concluded that procedural learning is impaired in dyslexia, consistent with the procedural deficit hypothesis (Ullman, 2004; Nicolson and Fawcett, 2007). Furthermore, they suggested that dyslexia might be associated with an overcompensation on the part of declarative memory and that declarative memory-based compensation might occur to a greater extent in older adults, due to declarative memory improving over time. Note, that this evidence was collected only from studies that incorporated the SRT task paradigm and hence does not speak to performance of children and adults with dyslexia on other types of tasks (i.e., AGL, Hebb, etc.); however, the findings do suggest that statistical learning is impaired in dyslexia, at least for the SRT task.

A second meta-analysis by van Witteloostuijn et al. (2017) focused exclusively on studies that evaluated visual artificial grammar learning in dyslexia. They found evidence of impaired learning in dyslexia with an effect size of 0.46. Interestingly, akin to Lum et al. (2013), their results also indicated an age effect, with a larger effect in children compared to adults. However, van Witteloostuijn et al.’s (2017) analysis also suggested the presence of publication bias, which calls these effects into question.

Finally, Schmalz et al. (2017) provided a systematic review of learning in dyslexia as assessed by both the SRT and artificial grammar learning tasks. Like van Witteloostuijn et al. (2017), they note the presence of publication bias and conclude that there is “insufficient high-quality data to draw conclusions.”

Thus, as is evident from these three metanalytical reviews, the extent of a statistical learning impairment in dyslexia remains unclear. Although Lum et al.’s (2013) analysis is suggestive of such an effect, the two more recent reviews above raise the issue of publication bias, which calls this conclusion into question. Having used these three meta-reviews as a springboard, the aim of the next section is to examine statistical learning in dyslexia by focusing on studies published after the three meta-analyses and reviews, which also incorporate a broader range of tasks than just SRT and AGL. Because two of the reviews suggested the presence of age effects, we furthermore examine the studies for children versus adults separately.

## Statistical Learning in Dyslexia: Recent Empirical Evidence

**FIGURE 3:**
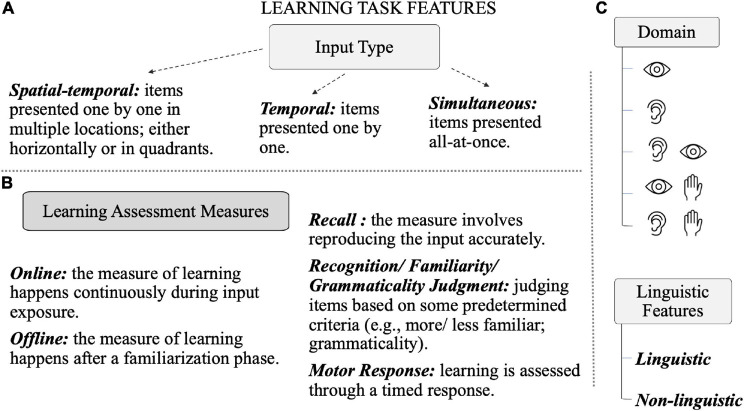
Common task features of statistical learning paradigms. **(A)** Schematic of different input types commonly used in tasks, each is accompanied by a short description. **(B)** Different learning assessment measures used in statistical learning tasks along with short descriptions. **(C)** Sensory domains from top to bottom: visual, auditory, auditory-visual, visual-motor, auditory-motor; and two types of task stimulus features: linguistic or non-linguistic.

Tables 1, 2 list the studies included in this review. All studies examine statistical learning (broadly construed to include any task assessing the learning of patterns) in children and adolescents (Table 1) and adults (Table 2) with dyslexia. Notably, none of these studies were *extensively* discussed in either of the three meta-analyses described earlier, or recent reviews on the current topic (Schmalz et al., 2017; Sawi and Rueckl, 2019; Bogaerts et al., 2020). We summarize the findings for each age group below. This section is followed by a more integrated discussion of findings. In order to highlight differences in task designs that may influence learning outcomes, Figure 3 provides a summary of task features that are referred to in the tables, such as the type of stimulus input (e.g., temporal, simultaneous), learning assessment measures (e.g., online-motor response), and other stimulus features (linguistic vs. non-linguistic; sensory domains, etc.).

**TABLE 1:** Table of studies with child participants; studies with more than one task are indicated by task number; tasks showing instances of impaired learning (top) are followed by ones showing intact learning (bottom).

Reference	Task no.	∼Age TD	∼Age DD	Task type	Input type	Domain	Linguistic features	Learning assessment		Language
**Impaired learning**										
He and Tong (2017)	1	CA:10; 5, RL:8; 4	10; 8	SRT	ST4	VM	NL	Online	Motor response	Can
Hedenius et al. (2020)	1	11; 7	11; 6	A-SRT	ST4	VM	NL	Online	Motor response	Swe
Schiff et al. (2017a)	1.1	CA:11; 9, RL:8; 5	11; 7	AGL: High complexity	S	V	NL	*Offline*	Gram judgment	Heb
Singh et al. (2018)	1	9; 4	10; 7	Predictor-target	T	VM	NL	Online	Motor response; EEG	Eng
Tong et al. (2019)	1	7; 7	7; 8	Embedded pattern	T	V	NL	*Offline*	Fam judgment	Can
Vandermosten et al. (2019)	1.2	Un:8; 9/Bi: 9*	8; 7	Non-native sound identification	T	AV	L	*Offline*	Recognition	Dut
**Intact learning**										
Staels and van den Broeck (2015)	2.1	10; 41	10; 76	Hebb: Digit sequencing	T	V	L	Online	Recall	Dut
Staels and van den Broeck (2015)	2.2	10; 41	10; 76	Hebb: Corsi blocks	ST	V	NL	Online	Recall	Dut
Staels and van den Broeck (2015)	2.3	10; 41	10; 76	Hebb: Abstract forms	T	V	NL	Online	Recall	Dut
Staels and van den Broeck (2017)	3.1	10; 3	10; 7	SRT implicit	ST2	VM	NL	Online	Motor response	Dut
Staels and van den Broeck (2017)	3.2	10; 3	10; 7	SRT explicit	ST2	VM	NL	Online	Motor response	Dut
Staels and van den Broeck (2017)	3.3	10; 3	10; 7	Contextual cueing implicit	S	VM	NL	Online	Recognition	Dut
Staels and van den Broeck (2017)	3.4	10; 3	10; 7	Contextual cueing explicit	*S*	VM	NL	Online	Recognition	Dut
Schiff et al. (2017a)	1.2	CA:11; 9, RL:8; 5	11; 7	AGL: Low complexity	S	V	NL	*Offline*	Gram judgment	Heb
Inácio et al. (2018)	1	CA&RL; 6–8	CA&RL; 6–8	AGL	S	V	NL	*Both*	Gram judgment, motor response	Por
van der Kleij et al. (2018)	1.1	11	11; 4	SRT	ST2	VM	NL	Online	Motor response	Dut
van der Kleij et al. (2018)	1.2	11	11; 4	Contextual Cueing	S	VM	L	Online	Motor response	Dut
Vandermosten et al. (2019)	1.1	Un:8; 9/Bi: 9*	8; 7	Native sound identification	T	AV	L	*Offline*	Recognition	Dut
van Witteloostuijn et al. (2019)	1	9; 8	9; 10	SRT	ST4	VM	NL	Online	Motor response	Dut
van Witteloostuijn et al. (2019)	1.2	9; 8	9; 10	Embedded pattern	T	VM	NL	*Both*	Motor response; recall, fam judgment	Dut
van Witteloostuijn et al. (2019)	1.3	9; 8	9; 10	Non-adjacent embedded pattern	T	AM	L	*Both*	Motor response; recall, gram judgment	Dut
West et al. (2019)	1.1	7; 6	9; 8	SRT	ST4	VM	NL	Online	Motor response	Eng
van Witteloostuijn et al. (2021a)	1.1	9; 8	9; 10	SRT	ST4	VM	NL	Online	Motor response	Dut
van Witteloostuijn et al. (2021a)	1.2	9; 8	9; 10	Embedded pattern	T	V	NL	*Offline*	Recall, fam judgment	Dut
van Witteloostuijn et al. (2021b)	1.1	9; 8	9; 10	SRT	ST4	VM	NL	Online	Motor response	Dut
van Witteloostuijn et al. (2021b)	1.2	9; 8	9; 10	Non-adjacent embedded pattern	T	AM	L	Online	Motor response	Dut

*Typical readers were assigned to a group exposed, either to a unimodal (Un) or a bimodal (Bi) speech sound distribution. Age: CA, chronological age; RL, reading level; Task type: SRT, serial reaction time; A-SRT: alternating serial reaction time; AGL: artificial grammar learning; Input type: T, temporal; S, simultaneous; ST2, 2 spatial-temporal locations; ST4, 4 spatial-temporal locations; Groups: TD, typically developing; DD, developmental dyslexia; Domain: V, visual; VM, visual-motor; AM, auditory-motor; AV, auditory-visual; Stimuli: L, linguistic; NL, non-linguistic; Learning assessment: gram, grammaticality; fam, familiarity; Language: Can, Cantonese; Dut, Dutch; Eng, English; Heb, Hebrew; Swe, Swedish. Both: online and offline measures.

**TABLE 2:** Table of studies with adult participants; studies with more than one task are indicated by task number; tasks showing instances of impaired learning (top) are followed by ones showing intact learning (bottom).

Reference	Task no.	∼Age TD	∼Age DD	Task type	Input type	Domain	Linguistic features	Learning assessment		Language
**Impaired learning**										
Bogaerts et al. (2015)	1.1	21;3	20;6	Hebb only	T	V	L	Online	Recall	Dut
Bogaerts et al. (2015)	1.2	20;2	21;3	Hebb + Hebb-pause detection	T	VM	L	Online	Recall	Dut
Gabay et al. (2015)	1.1	22;1	21;5	Embedded pattern	T	A	L	*Offline*	Fam judgment	Eng
Gabay et al. (2015)	1.2	22;1	21;5	Embedded pattern	T	A	NL	*Offline*	Fam judgment	Eng
Kahta and Schiff (2016)	1.1	18–33	18–33	AGL - implicit	S	V	L	*Offline*	Gram judgment	Heb
Henderson and Warmington (2017)	1.1	20;3	21;1	Hebb	T	A	L	Online	Recall	Eng
Henderson and Warmington (2017)	1.3	20;3	21;1	Finger tapping	T	*manual*	NL	Online	Motor response	Eng
Schiff et al. (2017b)	1.2	24;8	24;7	AGL implicit (with feedback)	T	A	NL	*Offline*	Gram judgment	Heb
Sigurdardottir et al. (2017)	1	26;3	26;8	Embedded pattern	ST4	V	NL	*Offline*	Fam judgment	Ice
Kahta and Schiff (2019)	1	19–35	19–35	AGL	T	A	NL	*Offline*	Gram judgment	Heb
Dobó et al. (2021)	1	17:06	16:08	Embedded pattern	T	A	L	*Offline*	Fam judgment	Hun
**Intact learning**										
Kahta and Schiff (2016)	1.2	18–33	18–33	AGL - explicit	S	V	L	*Offline*	Gram judgment	Heb
Samara and Caravolas (2017)	1.1	20;5	20;8	AGL	S	V	NL	*Offline*	Gram judgment	Eng
Samara and Caravolas (2017)	1.2	20;5	20;8	AGL	S	V	L	*Offline*	Gram judgment	Eng
Schiff et al. (2017b)	1.1	23;3	23;9	AGL explicit (with feedback)	T	A	NL	*Offline*	Gram judgment	Heb
Henderson and Warmington (2017)	1.2	20;3	21;1	SRT	ST4	VM	NL	Online	Motor response	Eng
Staels and van den Broeck (2015)	1.1	21;64	20;68	Hebb: Verbal	T	V	L	Online	Recall	Dut
Staels and van den Broeck (2015)	1.2	21;64	20;68	Hebb: Verbal	T	A	L	Online	Recall	Dut
Staels and van den Broeck (2015)	1.3	21;64	20;78	Hebb: Dots	ST4	V	NL	Online	Recall	Dut

*Task type: SRT, serial reaction time; AGL: artificial grammar learning; Input type: T, temporal; S, simultaneous; ST4, 4 spatial-temporal locations; Groups: TD, typically developing; DD, developmental dyslexia; Domain: V, visual; VM, visual-motor; A, auditory; Stimuli: L, linguistic; NL, non-linguistic; Learning assessment: gram, grammaticality; fam, familiarity; Language: Dut, Dutch; Eng, English; Heb, Hebrew; Hun, Hungarian; Ice, Icelandic.*

### Children

An inspection of Table 1 reveals that of the 26 separate tasks involving children with dyslexia (across 14 studies), only six showed impairment in statistical learning (He and Tong, 2017; Schiff et al., 2017a; Singh et al., 2018; Tong et al., 2019; Vandermosten et al., 2019; Hedenius et al., 2020), whereas the other 20 reported intact learning (Staels and van den Broeck, 2015, 2017; Schiff et al., 2017a; Inácio et al., 2018; van der Kleij et al., 2018; van Witteloostuijn et al., 2019, 2021a,b; Vandermosten et al., 2019; West et al., 2019). Interestingly, a majority of the studies with children were administered to native speakers of Dutch (Staels and van den Broeck, 2015, 2017; van der Kleij et al., 2018; Vandermosten et al., 2019; van Witteloostuijn et al., 2019, 2021a,b), of which all showed evidence of intact learning, except for performance on one of the tasks by Vandermosten et al. (2019). Besides Dutch and English (Singh et al., 2018; West et al., 2019), tasks were also administered to native speakers in other languages, such as Cantonese (He and Tong, 2017; Tong et al., 2019), Hebrew (Schiff et al., 2017a), Portuguese (Inácio et al., 2018) and Swedish (Hedenius et al., 2020). Continuing with this preliminary evaluation of studies listed in Table 1, certain task features tended to dominate over others. For example, there were more tasks pertaining to the visual and visual-motor domains versus the auditory-visual (Vandermosten et al., 2019) and auditory-motor (van Witteloostuijn et al., 2019, 2021b). This modality issue is discussed further in Section “Discussion.” In addition, most tasks involved temporally presented input, rather than simultaneous spatial arrays. Of the nine tasks examining SRT learning, only two showed impairment (He and Tong, 2017; Hedenius et al., 2020). However, two other studies that also employed overnight consolidation found intact learning on the SRT task (Inácio et al., 2018; van der Kleij et al., 2018). Thus, while the previous meta-analysis examining the SRT task in dyslexia concluded that a learning impairment does exist and was greater for children than in adults (Lum et al., 2013), based on the studies reviewed here, it is difficult to draw that same conclusion, especially considering the possibility of publication bias (Schmalz et al., 2017).

On a first pass of Table 1, there does not appear to be a clear pattern of results that would explain why certain studies found a statistical learning impairment in children and others did not. To examine this issue further, we elaborate first on the studies with *impaired* performance followed by a similar review of findings centered on *intact* performance.

Hedenius et al. (2020), for instance, reported impaired learning measured by RTs using an alternating SRT. They found that performance (i.e., the learning effect) for the dyslexia group did not improve with either time and exposure/practice, or consolidation as was the case for those without dyslexia. In contrast, He and Tong (2017) used a modified SRT, but unlike Hedenius et al. (2020), had two control groups (reading and age matched) to compare with the dyslexia group. He and Tong (2017) found that children with dyslexia exhibited a significant learning effect in the condition with a higher number of exposures compared to the one with lower number of exposures (in which no significant learning effect was found). Thus, learning was only impaired with less exposure to the sequence; additionally, those with dyslexia were overall slower than both reading controls. Also, unlike He and Tong (2017), Hedenius et al. (2020) did not account for overnight consolidation. Another point regarding methodology, is that in He and Tong (2017), a reward scheme was introduced after every block, which might have affected participant motivation across groups, differentially.

In one of few studies examining the effects of task complexity on statistical learning, Schiff et al. (2017a) demonstrated that grammar complexity (or topological entropy; see Bollt and Jones, 2000) matters while comparing children with and without dyslexia. They used a high complexity grammar to investigate whether chunk strength and grammar system complexity would influence grammaticality judgment. Not only was performance below chance on grammatical accuracy but also on classification in those with dyslexia (compared to reading and chronological age matched controls). We return to the issue of task complexity, in Section “Discussion.”

Singh et al. (2018) was the only study in Table 1 with children to incorporate neurophysiological measures (i.e., electroencephalography) to index statistical learning. They found that while children with dyslexia had atypical learning as reflected in the event-related potential waveforms, their behavioral performance was comparable to typical controls. This is an important finding as it shows a potential discrepancy in the assessment of learning via RTs (which may reflect a type of implicit motor learning, see Batterink et al., 2015) vs. recordings from this particular event-related brain signal (which may reflect attention-based learning, see Singh et al., 2017). This finding resonates with the view that statistical learning involves both implicit and explicit/controlled aspects of learning. It also highlights that neural measures of learning may provide additional information not captured solely by behavioral measures.

Tong et al. (2019) reported impaired learning on both a visual embedded pattern triplet task (similar to that used by Arciuli and Simpson, 2011) as well as orthographic awareness (not discussed here). In the embedded pattern learning task, the inability to acquire the contingencies between the ordered non-linguistic, visual stimuli, suggests that dyslexia is not just associated with a phonological deficit, but a more general learning impairment.

A final study showing impairment found that when children with dyslexia were presented with native (Dutch) and non-native (Hindi) sounds, they did not utilize embedded statistical cues to learn phoneme differences, unlike the children without dyslexia, who did (Vandermosten et al., 2019). This resulted in less distinct phonemic categories which thus subsequently (negatively) affected their ability to connect between these sounds and their written representation. These findings could potentially be interpreted by the perceptual noise exclusion theory (Sperling et al., 2005) and/or the anchoring deficit theory (Banai and Ahissar, 2018) both of which suggest poor sensitivity to tracking the distribution of sound statistics in individuals with dyslexia.

Turning now to studies showing *intact* learning, Staels and van den Broeck (2015) reported no group differences between those with and without dyslexia on a variety of Hebb tasks, a finding that persisted, even after attentional functioning was accounted for. Likewise, no group differences were found with both implicit and explicit versions of the SRT and contextual cueing tasks (Staels and van den Broeck, 2017). In the explicit versions of these tasks, participants were informed about the existence of the statistical regularities but in the implicit versions, no such instructions were provided. Although no learning impairments were observed, the children with dyslexia exhibited slower RTs and lower accuracy overall.

In addition to the high complexity grammar mentioned above, Schiff et al. (2017a) also investigated whether knowledge at exposure could be generalized using a low complexity grammar. They found that both groups were able to use chunk knowledge as well as grammatical rules but to different degrees. Thus, although typical learners benefited mainly from chunk strength (high or low), children with dyslexia were primarily influenced by grammar complexity level, chunk strength being secondary. Taken together with their earlier task (Schiff et al., 2017a; high complexity grammar), the results suggest dyslexia may be associated with differences in learning pattern information, which might not manifest as a learning impairment *per se* unless using a more complex grammar (see also Schiff and Katan, 2014).

Inácio et al. (2018) measured artificial grammar learning over multiple sessions. During the third session (only), a distraction task was administered after which participants performed a grammaticality judgment task, thus obtaining a measure of grammaticality classification during which participants were instructed to respond ‘quickly.’ No differences in learning were observed, despite overall slower RTs in the dyslexia group. Accuracy scores also showed intact learning. Inácio et al.’s (2018) interpretation of comparable learning across groups was based on: reasonable effect sizes and overall performance. However, apart from group level analyses, an individual (*d’*) grammatical discrimination index based on classification accuracy revealed that only a few high performers in each group showed good discriminability but the majority, irrespective of group, performed at or below chance. Additionally, there was no significant correlation between this *d’* index and the accuracy with which participants could later reproduce the previously viewed sequences using a set of cards with geometric figures. The fact that so many individuals did not appear to show a learning effect could be indicative of issues with the task itself.

In a large set of studies, van Witteloostuijn et al. (2019, 2021a, 2021b) showed that learning was intact across tasks, whether they were SRT, visual statistical learning, or auditory non-adjacent learning. The lack of strong group differences despite presenting stimuli in visual-motor and auditory-motor domains across a variety of tasks is quite telling (van Witteloostuijn et al., 2019, 2021a). Similarly, van der Kleij et al. (2018) also reported no group differences after testing children with both SRT and spatial contextual cueing tasks on each of 2 days. Interestingly, resonating with some of the previously reviewed studies, participants with dyslexia had overall longer RTs. Likewise West et al. (2019), also reported no overall significant group differences in learning on the SRT task. However, they found that the dyslexia group showed initial poor learning relative to controls, though they continued to show learning throughout the task.

Thus, although the majority of studies demonstrated a lack of group differences, more subtle differences in performance were observed, such as overall increased RTs for those with dyslexia compared to typical readers. In other words, the lack of a detectable group difference need not mean that statistical learning is comparable across groups. In fact, slower RTs could indicate a temporal processing or visual-spatial attention deficit, even though overall no statistically significant group differences on the primary measures of learning were observed. It is therefore possible that for some tasks, the ways of assessing learning were not sensitive enough to detect group differences and may only become apparent with more complex input patterns (e.g., Schiff et al., 2017a) or the use of neural measures (e.g., Singh et al., 2017). Individual variability within each group is also important to examine more closely (e.g., Inácio et al., 2018), as pointed out by Arciuli and Conway (2018).

To summarize, the studies reviewed here do not provide strong evidence for a statistical learning impairment in children with dyslexia. However, several potential areas to follow up are the role of stimulus complexity (Schiff et al., 2017a) overnight consolidation (Hedenius et al., 2020), and whether there exists a generalized information processing delay as indicated by longer RTs in some studies (van der Kleij et al., 2018; West et al., 2019). Furthermore, it may be important to not only rely on behavior but also to include neural measures (e.g., Singh et al., 2018; see also for instance Menghini et al., 2006). Finally, more work is needed examining individual differences to help understand heterogeneity of abilities within dyslexia (Inácio et al., 2018). Some of these points will be elaborated upon in Section “Discussion” below, after we review the adult studies.

### Adults

Of the 19 separate tasks involving adults (across 10 studies), 11 of them showed impaired learning in dyslexia (Bogaerts et al., 2015; Gabay et al., 2015; Kahta and Schiff, 2016, 2019; Henderson and Warmington, 2017; Schiff et al., 2017b; Sigurdardottir et al., 2017; Dobó et al., 2021). The impairments were observed for a variety of task types, including linguistic (both auditory and visual) versions of the Hebb task (Bogaerts et al., 2015; Henderson and Warmington, 2017), linguistic and non-linguistic auditory versions of the SRT task (Gabay et al., 2015), non-linguistic auditory versions of the artificial grammar learning task (Kahta and Schiff, 2016; Schiff et al., 2017b), and a non-linguistic visual embedded pattern task (Sigurdardottir et al., 2017). Thus, in contrast to the findings with children, these studies are more consistent with a global, domain-general statistical learning impairment in adults with dyslexia.

However, intact statistical leaning was also found across task types (Staels and van den Broeck, 2015; Kahta and Schiff, 2016; Henderson and Warmington, 2017; Samara and Caravolas, 2017; Schiff et al., 2017b). Interestingly, intact learning was reported for a majority of studies involving a simultaneous presentation in a finite state grammar paradigm (Kahta and Schiff, 2016; Samara and Caravolas, 2017). Studies with adults were administered in many different native languages, though no one language was predominantly represented as was the case with children (e.g., Dutch). For instance, adult participants spoke either Dutch (Bogaerts et al., 2015; Staels and van den Broeck, 2015), English (Gabay et al., 2015; Henderson and Warmington, 2017), Hebrew (Kahta and Schiff, 2016, 2019; Schiff et al., 2017b) or Icelandic (Sigurdardottir et al., 2017).

As in the previous section, we elaborate first on the studies with *impaired* performance followed by a similar review of findings showing *intact* performance. Henderson and Warmington (2017) found partially impaired (significant initial effect that attenuated over time) statistical learning via an auditory-linguistic Hebb repetition task. Additionally, to rule out whether any observable group differences were due to poor motor control, Henderson and Warmington (2017) also administered a motor tapping (non-sequential) task to all participants and concluded that those with dyslexia did in fact show a significantly slower processing speed than adults without dyslexia.

In another set of Hebb learning studies, Bogaerts et al. (2015) reported that adults with dyslexia initially exhibited impaired learning and need significantly more exposure to repetitions (than controls) on a Hebb task to reach a pre-established learning criterion. Some of these adults, however, (although not all) eventually developed long-term serial order representations, over time. Adults with dyslexia also had poorer (relative to controls) baseline performance on fillers, suggesting worse memory capacity, for serial order. Retention (in terms of memory savings over time), however, became comparable across groups after 1 month of initial learning. Even after controlling for baseline differences across groups (average filler performance), the finding of impaired serial order learning persisted. Additionally, once learned, though initially slow, both groups showed comparable retention on Hebb sequences, after consolidation (of 1 day; and even after 1 month). In their second experiment (Hebb learning and pause detection), Bogaerts et al. (2015) sought to replicate their first experiment but they also measured lexical engagement by introducing lexical competition in Hebb sequences via a pause detection task (for a more nuanced description see Szmalec et al., 2012 and Bogaerts et al., 2015). To measure lexical engagement, lexical competition is expected to occur when participants are presented with Hebb sequence containing base words (e.g., *lavabu*) that would interfere with words form the participants’ (Dutch) mental lexicon (e.g., *lavabo*, meaning kitchen sink). Thus, participants when presented with these sequences, both with and without an artificially embedded pause had to quickly indicate the presence (or not) of a pause. Successful detection of the pause would mean selecting from amongst multiple candidates in the mental lexicon, depending on knowledge of recently learned Hebb sequences (containing lexical neighbors). Bogaerts et al. (2015) concluded that lexical engagement (interaction of new items with pre-existing word-forms in the mental lexicon) did occur for control participants but was less robust for adults with dyslexia, even after 1 month, although this effect was not strongly (statistically) supported. When combined, results indicate that adults with dyslexia were impaired in the long-term learning of verbal serial order information and also showed weaker lexicalization of novel word forms.

Notably, the predecessor to both Szmalec et al. (2011), Bogaerts et al. (2015), and Henderson and Warmington (2017), who also showed Hebb learning impairment for individuals with dyslexia, administered three Hebb versions: a verbal-visual, verbal- auditory and visuospatial version; and reported poor learning amongst those with dyslexia for all three task versions. These findings in adults contrast with the relatively intact Hebb learning in children. It is thus possible that there may be developmental and cognitive changes in adulthood that account for the impaired Hebb learning across sensory domains.

In another study indicating impaired learning, Gabay et al. (2015) incorporated speech syllables used by Saffran et al. (1996) that were presented to participants during a passive familiarization. At test, they then had to respond by judging which one of a pair (word and part-word) was similar to sounds in familiarization, via a two alternative forced choice response option. Transition probabilities varied from 1.0 within a word (hearing da after bi in bidaku) to <1.0 between words (probability of encountering any of the beginning syllables in padoti, gulabu or tupiro presented after ku in bidaku goes down to 0.33). Similarly, they also had a non-speech version of the task in which they used non-linguistic tones that were from Saffran et al. (1999). After continuous temporal presentation (exposure) of the two types of stimuli, participants then had to complete a familiarity judgment at test. Relative to the control group, Gabay et al. (2015) reported impairment for those with dyslexia, across both speech and non-speech materials, despite overall above chance performance.

Similarly, Sigurdardottir et al. (2017), used an embedded pattern task with a spatio-temporal presentation, but resorted to offline, rather than online-motor response acquisition. Sigurdardottir et al.’s (2017) findings were nonetheless similar to those of Gabay et al. (2015), revealing that unlike typical learners, those with dyslexia had trouble with familiarity judgment (at test) of shape pairs (nonsense letter-like stimuli) they had viewed previously during familiarization. Thus, the nature of the learning impairment appears consistent across linguistic and non-linguistic patterns, at least for tasks with embedded patterns.

Turning to a visual artificial grammar paradigm, Kahta and Schiff (2016) also reported group differences. Performance of those without dyslexia exceeded chance under both implicit and explicit learning conditions (where participants were or were not informed of embedded rules at training). However, in the dyslexia group, impairment was reported for implicit learning but was intact on the explicit version of the task. Keeping the same paradigm but changing the sensory modality, Schiff et al. (2017b) investigated the effects of feedback on implicit and explicit learning with auditory stimuli. Despite a decrease in false alarms across groups, feedback under implicit conditions lead to increased hits only for those without dyslexia; however, under explicit conditions, feedback was more helpful to those with dyslexia.

Kahta and Schiff (2019) similarly investigated artificial grammar learning under implicit and explicit conditions, using temporally presented tones. They found that results in the auditory modality were similar to the implicit condition visual findings of Kahta and Schiff (2016), in that although both groups performed above chance, *d’* values were significantly lower for those with dyslexia compared to controls, linking such performance to an anchoring deficit (Ahissar, 2007). On the other hand, auditory modality findings showed comparable performance across groups under explicit learning conditions. Together, these findings suggest a complex interplay between the presence of instructions and feedback and how these factors interact to influence the learning of visual and auditory patterns dyslexia. More work is needed to clarify these research findings.

In addition to the studies above, only one study examined statistical learning in adolescents (Dobó et al., 2021), with a report of impaired learning for the dyslexia group on an auditory statistical learning task. More work is needed to follow-up on a potential auditory statistical learning impairment in adolescents. Currently, it is difficult to conclude based on this study alone, that adolescents with dyslexia are impaired on statistical learning.

Turning next to studies showing intact learning, two of these are already discussed above in tandem with the impaired findings. In short, Kahta and Schiff (2016), found impaired learning in the implicit version of their artificial grammar learning study, compared to relatively intact *explicit* learning. Similarly, Schiff et al. (2017b), in addition to the points above, showed that children with dyslexia had more false alarms than controls under implicit learning but they did benefit more from feedback in the explicit condition.

Samara and Caravolas (2017) concluded that adults with dyslexia learned grammaticality and chunk strength knowledge that was comparable to typical readers on both a linguistic as well as a non-linguistic version of the same artificial grammar learning task. Notably, Samara and Caravolas (2017) explicitly instructed participants to both memorize, as well as recall each letter string following exposure, which may have impacted learning outcomes. Additionally, because Samara and Caravolas (2017) excluded some participants based on their clinical profile (due to comorbid diagnoses), their study was unable to ascertain if a more heterogeneous group of individuals would have been more likely to show impaired learning. One thing to note is that the artificial grammar learning paradigm typically only incorporates offline measures of learning, which may mask more subtle differences in the learning profiles between typical and atypical readers that could only be detected with online measures.

In addition to the impaired Hebb and manual-motor learning described above, Henderson and Warmington (2017) also found overall intact learning in a SRT. Further, they reported that the dyslexia group tended to have longer RTs and more variability compared to controls, which echoes other indications of increased RTs in children, as reviewed in the previous section. Overall, both groups showed evidence of a learning effect in terms of RTs and accuracy (significantly higher for sequenced vs. random trials) as well as in terms of consolidation (both groups showed sustained learning that grew stronger with time).

In line with their findings in children, but in contrast with previous work on Hebb learning impairments in dyslexia (e.g., Szmalec et al., 2011; Henderson and Warmington, 2017). Staels and van den Broeck (2015) did not report Hebb learning deficits for adults with dyslexia in any modality (verbal-visual, verbal-auditory, non-verbal visuo-spatial).

To summarize, compared to research on children, relatively more tasks found learning impairments in adults with dyslexia, across a variety of task types and learning conditions. This result is in contrast to two previously described meta-reviews which concluded there was a greater impairment in children with dyslexia relative to adults (Lum et al., 2013; van Witteloostuijn et al., 2017). However, whether the current findings of learning impairment in adults is an actual effect or due to publication bias remains to be seen, and is an issue that will be taken up in greater detail next.

## Discussion

Based on our review of recent studies, we first offer suggestive trends in the findings that warrant closer inspection, followed by suggestions for future research. We close by addressing the extent to which the evidence supports a statistical learning deficit in dyslexia.

### Statistical Learning in Dyslexia: Suggestive Trends

#### Task Heterogeneity

Although the tasks included in this review are all taken to index statistical learning, there are many differences across tasks that complicate interpretation of the findings. Tasks differed in terms of input type (e.g., temporal, simultaneous, etc.), sensory domain (e.g., visual, auditory, etc.), whether the stimuli were linguistic or non-linguistic, how learning was measured, and whether the participants were allowed overnight consolidation before a final test session. As has been pointed out recently, it is possible that each type of task may be tapping into a different aspect of statistical learning (Arciuli and Conway, 2018; Bogaerts et al., 2020). It is currently unclear how tasks map onto different learning constructs. Taking a neural perspective might help in this regard. For instance, it is now fairly well-established that the SRT task is heavily dependent upon basal ganglia circuitry (e.g., Doyon et al., 2009; Ullman et al., 2020), whereas the embedded patterns task seems to involve a combination of neocortical sensory networks and possibly the hippocampus (Frost et al., 2015; Conway, 2020). These differences at the neural level likely in turn have implications for the type of cognitive algorithm or mechanism that is being brought to bear for any given task, such as declarative vs. procedural memory (Sawi and Rueckl, 2019), chunking vs. learning of transitional probabilities (Perruchet and Pacton, 2006), or attention-dependent vs. attention-independent modes of learning (Conway, 2020).

#### Age Effects

Whereas findings from recent meta-analyses suggest that statistical learning impairments are more pronounced in children, our review of recent studies suggests the opposite conclusion. Task heterogeneity and possibly even differences in participant characteristics could be behind this discrepancy. From the current review, impairments were more frequently observed in studies with adults relative to children, at least for the Hebb repetition learning task. One explanation for the age effects observed with the SRT task in earlier meta-analyses is that it is possible that adults with dyslexia (relative to children) are able to compensate for procedural memory deficits by using their declarative memory system (Lum et al., 2013). However, this same explanation might not explain the opposite age effects observed with the Hebb task because unlike performance on the SRT, performance on the Hebb task has not been strongly associated with procedural learning (Nicolson and Fawcett, 2007). In fact, the Hebb task has been associated with modulation of brain regions such as the hippocampus, cingulate cortex and inferior frontal cortex (Attout et al., 2020), making it more likely to be a form of declarative memory or potentially relying on working memory rather than procedural memory. Thus, a greater Hebb learning impairment in adults rather than children with dyslexia doesn’t seem to be explained well by a compensatory mechanism of declarative memory. Instead, greater learning impairment in adults could be because different tasks may be tapping into different cognitive mechanisms that may have different developmental trajectories. For instance, implicit forms of statistical learning might be relatively age-invariant, whereas learning that involves top-down attentional control likely increases with age (Conway, 2020). If performance on the Hebb (and similar) task(s) does in fact benefit from top-down attentional control, which could be less well developed in adults with dyslexia, it would explain the inferior performance on these tasks relative to neurotypical adults. In sum, the potential age effects observed here are based on a small set of studies and therefore deserves more attention and study.

#### The Role of Publication Bias

As previously mentioned, recent metanalytical reviews (Schmalz et al., 2017; van Witteloostuijn et al., 2017) have reported a publication bias in studies with dyslexia, at least with the SRT and artificial grammar learning tasks. Bias generally occurs when effect sizes are unevenly distributed within the literature, potentially due to underreporting of representative outcomes (because non-significant or less interesting results are less likely to be published, Type I error) or if a disproportionate number of studies are underpowered (Type II errors), thus obscuring the true effect size of the sample (Egger et al., 1997). The bias or skew creates a disconnect between the actual phenomenon under study and the way it is represented in the literature. As many have suggested (Nosek et al., 2018, 2019), one way to reduce publication bias is to promote pre-registration of studies such that the nature of planned analyses (*a priori* vs. *post hoc*) are documented ahead of time. This approach is intended to minimize any selective reporting, *post hoc*, on the part of the researcher because a plan of action was already predetermined. Most importantly, registration encourages final publication, irrespective of study outcomes. While preregistration requires adequate planning, it is beneficial for dealing with publication bias (Nosek et al., 2019). We should point out that to the best of our knowledge, none of the studies discussed in our review were preregistered.

#### Other Possible Group Differences That May or May Not Indicate Impaired Statistical Learning

It was interesting to note that across a range of studies and paradigms, there was some indication that individuals with dyslexia may exhibit a general delay in RT measures (e.g., Henderson and Warmington, 2017; van der Kleij et al., 2018; West et al., 2019). This general slowing of RTs could be due to slower information processing at a cognitive level or simply slowed motor responding on a novel task requiring speed and accuracy; such effects have been previously observed in dyslexia using sequential motor tasks (Marchand-Krynski et al., 2017). Delayed information processing could have a cascade of effects on learning and processing, including potential issues with statistical learning. More research is needed to examine the existence and nature of such delay in RTs and how or if it relates to learning and reading impairment. Similarly, other cognitive processes need to be taken into account, that may or may not directly impact statistical learning, such as differences in attention, working memory, or executive functions in individuals with dyslexia.

#### Examining Individual Differences in Dyslexia

It should be noted that individual differences have largely been ignored in studies on statistical learning in dyslexia (Schmalz et al., 2017; Arciuli and Conway, 2018; Bogaerts et al., 2020). In addition to age which was discussed already, other individual variables could also play into the statistical learning – dyslexia link, including sex, degree of reading impairment, existence of comorbidities, cognitive factors such as attention and working memory, and even personality traits such as openness to experience (Kaufman et al., 2010). Any such investigations are complicated by the fact that both statistical learning and reading ability are each driven by multiple sub-processes (Arciuli, 2017; Arciuli and Conway, 2018; Conway, 2020) and a break-down could occur at any level of processing. The typical learning measurements rarely capture equivalent, homogenous constructs across tasks, and similarly dyslexia seldom constitutes a single learning profile. Thus, individual differences may prove valuable in future studies to better understand the nature of variability in both statistical learning tasks as well as the dyslexia profile.

#### The Importance of Measuring Overnight Consolidation

Studies involving consolidation before a subsequent learning assessment are important for helping to advance our current understanding of the role of sleep and memory in statistical learning and how it might differ in individuals with dyslexia. For instance, some studies (e.g., Bogaerts et al., 2015; Henderson and Warmington, 2017; Inácio et al., 2018) assessed learning at more than one time-point, thereby providing evidence of learning as a trajectory rather than on a single occasion. Overnight consolidation specifically is known to aid learning of sequential information (Janacsek and Nemeth, 2012) and therefore is important to examine in dyslexia. However, as Janacsek and Nemeth (2012) indicate, consolidation can be sleep dependent or sleep-independent, depending on whether learning in the task is explicit or implicit, respectively (Robertson, 2009). Thus, the role of consolidation on statistical learning in those with and without dyslexia deserves further exploration.

### Future Directions

#### Task Complexity

Aside from the work by Schiff et al. (2017a), one area that has not been explored in detail is the effect of task complexity. For example, the complexity of artificial grammars affects learning in typical readers (Schiff and Katan, 2014). It is possible, for instance, that individuals with dyslexia may show increased difficulty learning more complex grammars or patterns, but this has only rarely been directly examined. As pointed out earlier, whereas Schiff et al. (2017a) found impaired learning in children with dyslexia using an artificial grammar with high levels of complexity, intact learning was found when using lower levels of complexity. In addition to grammar complexity, other task features, such as the length of sequences, the amount of exposure to the input, whether the task involves learning a recurring pattern or generalizing a pattern to new stimuli, all could be influencing task performance. The role of complexity (and the presumed increase in task demands) is important to take into consideration, especially as it may impact working memory, attention, and even motivation, across tasks.

#### Visual Modality Task Dominance

The majority of studies used visual or visual-motor paradigms rather than auditory, a trend that is also apparent in the list of studies reviewed by Bogaerts et al. (2020) and Schmalz et al. (2017). One reason for this could be that one of the most well-studied tasks, the SRT task, is designed mainly for acquisition of visual-motor responses. Another commonly used task is the artificial grammar learning task, which traditionally incorporates visual stimuli (though conceivably any type of stimulus can be used). Future studies assessing learning in dyslexia would benefit from examining learning across modalities (i.e., auditory, visual, auditory-visual, auditory-motor and visual-motor). This will help us understand to what extent the degree of learning impairment in those with dyslexia is domain-general or is focused only on a specific type of stimulus or perceptual domain.

#### Neural Measures as a More Sensitive (or Additional) Indicator of Learning

It is possible that learning differences may be present in some individuals with dyslexia but that the measures used to assess learning are not sufficiently sensitive. Neural measures might provide such sensitivity. As mentioned earlier, Singh et al. (2018) observed intact learning in adults with dyslexia on a serial predictor-target task using RTs as a measure, but atypical learning effects using event-related potentials (ERPs). They concluded that the RT measures were indexing a more reactive, implicit form of learning, but the ERP responses were measuring attention-dependent, explicit learning (see also Batterink et al., 2015). Interestingly, the P3 was also reported by Fosker and Thierry (2004) linking the P3 with change detection of phonemes in adults with dyslexia. They found that unlike typical readers, those with dyslexia had a hard time with attention-shifting to a phonological cue. Thus, neural measures such as the P3 component might provide more sensitive measures for detecting learning differences between those with and without dyslexia.

#### Learning and Development

Multiple factors appear to affect how statistical learning develops across the lifespan, and it is currently unknown to what extent such developmental factors are altered due to dyslexia. Although statistical learning was initially considered to be age-invariant with learning observed in very young infants (e.g., Saffran et al., 1996), more recent studies suggest age-related learning effects for both auditory (Shufaniya and Arnon, 2018; Kidd et al., 2020) and visual statistical learning (Arciuli and Simpson, 2011; Raviv and Arnon, 2018). However, recent findings suggest that age effects appear to depend on whether task stimuli are linguistic or non-linguistic: Shufaniya and Arnon (2018) found that *non-linguistic* auditory statistical learning increased in children aged between 5 and 12 years whereas learning of linguistic auditory patterns was age-invariant (Raviv and Arnon, 2018). Complicating the picture, the type of learning assessment in the test phase may also interact with age effects. For example, Kidd et al. (2020) provided empirical evidence of linguistic auditory statistical learning increasing with age, but only when the learning assessment was based on statistically induced chunking recall (Isbilen et al., 2017) as opposed to more commonly used measures (e.g., perceptual judgment). Although these findings were largely limited to those without a neurodevelopmental issue, it may be relevant to future work involving dyslexia. Lastly, it should also be pointed out that very few studies have focused on adolescents (Dobó et al., 2021; Lukács et al., 2021); thus, more studies targeting this age group are needed.

#### Online vs. Offline Learning Measures

Finally, more attention ought to be paid to the effects of online versus offline measures of learning. For instance, Lukács et al. (2021) administered auditory-linguistic (verbal) and visual-non-linguistic (non-verbal) versions of tasks based on the SRT to native Hungarian speakers. Participants were adolescents with varying levels of reading proficiency. For both tasks, learning was tracked with online motor responses as well as offline familiarity judgments. While the offline measures showed learning only in the acoustic-verbal modality, online measures were more sensitive in capturing acoustic-verbal and visual-non-verbal learning over time. Thus, these and similar results contrasting both types of measures (Inácio et al., 2018; van Witteloostuijn et al., 2019), confirm that online vs. offline measures may be sensitive to different aspects of learning (see also Siegelman et al., 2018). This line of inquiry is worth following-up in future study designs as the use of different measures of learning may be more or less likely to reveal group differences.

## Is There a Statistical Learning – Dyslexia Link?

After reviewing the recent literature, the question remains, is there a statistical learning impairment in dyslexia? And more generally, what theory or combination of theories best accounts for the extant data? We believe that the evidence for a statistical learning impairment in dyslexia is not strong, but is suggestive, perhaps more so in adults than in children. Certainly, it does not appear to be the case that all individuals with dyslexia have a global statistical learning impairment for all tasks. It also does not appear to be the case that there is a single theoretical framework, out of all the various frameworks reviewed earlier, that clearly and sufficiently encompasses the findings reviewed here. Instead, it could be that a combination of theories together might account for some of the patterns of learning differences reviewed.

For instance, the phonological deficit could explain why tasks with visual-linguistic stimuli show a learning impairment (e.g., Bogaerts et al., 2015; Kahta and Schiff, 2016); however, it does not adequately account for why tasks without a phonological or language component also show impairment (e.g., Gabay et al., 2015; Schiff et al., 2017a,b; Sigurdardottir et al., 2017, etc.) Similarly, support for the temporal processing deficit can be found in studies using temporal or spatial-temporal stimuli (e.g., Gabay et al., 2015; Henderson and Warmington, 2017; Kahta and Schiff, 2019; Dobó et al., 2021); however, there were also many temporal tasks that did not show an impairment, and tasks with simultaneously presented stimuli that did. Likewise, procedural memory, which is traditionally measured with the SRT task, showed impairment in some studies (e.g., He and Tong, 2017; Hedenius et al., 2020) but not others (e.g., van Witteloostuijn et al., 2019; West et al., 2019). Thus, no single deficit theory seems to adequately account for the profile of reported results, at least not on its own. It could be that a combination of theories is needed to account for the data, such as a combined deficit of temporal processing and automaticity. Multiple deficit theories may have a better chance to capture the empirical data relative to single deficit theories alone.

Notably, unlike the single-deficit models, multiple deficit models might better account for issues of comorbidity with other disorders (Moll et al., 2020). We suggest that multiple-deficit theories that focus on the confluence of cognitive, behavioral, neurophysiological as well as environmental factors might be best suited for understanding the complex interplay of factors that lead to dyslexia (Pennington et al., 2012). Multiple-deficit models of dyslexia, in some ways, parallel the multi-componential nature of statistical learning. Thus, although the single deficit theories in part address statistical learning issues, they may not always be comprehensive enough to capture the heterogeneity within the deficit profile of all impaired readers. However, a central issue with this approach, whether the focus is on single or double deficits, is that it is still too broad. We suggest adopting an alternative framework, one that might better account for individual differences that are known to be present in statistical learning performance (Siegelman et al., 2017; Johnson et al., 2020).

Rather than characterize deficits broadly (e.g., a procedural memory deficit or a temporal processing deficit or even a statistical learning deficit), it could be that the deficit is specific to a certain aspect of processing, such as only at encoding, consolidation, or retrieval. Similarly, it could be that particular deficits are only present for certain individuals and during certain task conditions (such as, the presence or absence of feedback). It may be beneficial to design tasks that not only focus on a specific aspect of learning (e.g., statistical learning of auditory non-linguistic sequences), but that also delineates different aspects of processing that contribute to learning (e.g., encoding vs. retrieval). Thus, designing studies that have a strong theoretical motivation, with some link to a proposed deficit, is important for future neurodevelopmental research (e.g., Bogaerts et al., 2020).

Likewise, attention needs to be focused on the specific neurocognitive mechanisms underlying statistical learning and sequence processing such as those outlined by Conway (2020) and Dehaene et al. (2015) and how these mechanisms are brought to bear in various task designs. Ultimately, our understanding of the extent to which statistical learning impairment exists in dyslexia will depend on more fine-tuned investigations of the specific neurocognitive mechanisms that underlie different aspects of statistical learning. This requires more work in neurotypical individuals to understand how the various sub-processes of statistical learning proceeds in typical development, for different types of tasks and ways of measuring learning (Arciuli and Conway, 2018). Once such pivotal areas are uncovered, this will help inform the nature of statistical learning in dyslexia.

## Conclusion

To summarize, we conclude that a statistical learning deficit in some form is likely to exist in dyslexia but is not well captured due to variability in task design, uneven assessment methods used to assess such learning, and the heterogeneity of dyslexia itself. Several trends were reviewed such as those related to publication bias, age effects, task heterogeneity, and the lack of studies incorporating neural measures and individual differences; all require follow up in future research. Although current theoretical frameworks are helpful for explaining certain aspects of deficit in dyslexia, a more refined explanation of the learning and processing differences as it relates to dyslexia, is needed.

## Author Contributions

SS led the preparation of this article including the literature review and first draft of the manuscript. CC provided overall guidance and contributed to subsequent drafts and revisions. Both authors contributed to the article and approved the submitted version.

## Conflict of Interest

The authors declare that the research was conducted in the absence of any commercial or financial relationships that could be construed as a potential conflict of interest.

## Publisher’s Note

All claims expressed in this article are solely those of the authors and do not necessarily represent those of their affiliated organizations, or those of the publisher, the editors and the reviewers. Any product that may be evaluated in this article, or claim that may be made by its manufacturer, is not guaranteed or endorsed by the publisher.
